# Potential Impact of Oat Ingredient Type on Oral Fragmentation of Biscuits and Oro-Digestibility of Starch—An In Vitro Approach

**DOI:** 10.3390/foods8050148

**Published:** 2019-05-01

**Authors:** Amparo Gamero, Quoc Cuong Nguyen, Paula Varela, Susana Fiszman, Amparo Tarrega, Arantxa Rizo

**Affiliations:** 1 Instituto de Agroquímica y Tecnología de Alimentos (IATA-CSIC), 46980 Valencia, Spain; agamero@iata.csic.es (A.G.); atarrega@iata.csic.es (A.T.); arantxa.rizo@iata.csic.es (A.R.); 2 Nofima AS. NO-1433 Ås Norway; quoc.cuong.nguyen@nofima.no (Q.C.N.); paula.varela.tomasco@nofima.no (P.V.)

**Keywords:** particle size, in vitro oral fragmentation, oral phase of starch hydrolysis

## Abstract

The aim of the present study was to determine how variation in the biscuit matrix affects both the degree of in vitro fragmentation and the starch hydrolysis that occurs during the oral phase of digestion. Using three different oat ingredient types (oat flour, small flakes, and big flakes) and baking powder (or none), six biscuits with different matrices were obtained. The instrumental texture (force and sound measurements) of the biscuits was analyzed. The samples were then subjected to in vitro fragmentation. The particle size distribution and in vitro oral starch hydrolysis over time of the fragmented samples were evaluated. The results showed that the samples presented different fragmentation patterns, mainly depending on the oat ingredient type, which could be related to their differences in texture. The biscuits made with oat flour were harder, had a more compact matrix and showed more irregular fragmentation and a higher percentage area of small particles than those made with big oat flakes, which were more fragile and crumbly. The highest degree of starch hydrolysis corresponded to the biscuits made with flour. Conclusions: Differences in the mechanical properties of the biscuit matrix, in this case due to differences in the oat ingredient, play a role in the in vitro fragmentation pattern of biscuits and in the oral phase of starch hydrolysis.

## 1. Introduction

Oral processing during eating involves cyclic mechanical breakdown and insalivation to transform food into a bolus that is easy and safe to swallow [1]. The changes that the food undergoes during the oral phase are relevant, since they play a key role in sensory perception, as reflected by the number of existing studies dealing with the relationship between oral processing and texture perception [2] and flavor release [3]. The oral phase is also considered to have a great impact on digestion and nutrient absorption, despite its short duration compared with other phases of digestion, due to the food breakdown and insalivation that take place in the mouth [4,5,6].

A threshold of particle size and level of lubrication by saliva is required to form a well-lubricated, cohesive, ready-to-swallow bolus [3,7]. In starchy foods, such as biscuits, starch hydrolysis by salivary enzymes clearly begins in this in-mouth step [8] and depends on the initial structure and breakdown path of the food in the mouth. In turn, enzymatic hydrolysis of starch impacts the blood glucose response. Several studies on the impact of oral digestion have been conducted, both in vivo and in vitro [9,10].

At the same time, the number of studies of bolus properties has been increasing in recent years, providing insight about the physical and chemical changes that food products undergo during the oral phase [11]. Several methodological approaches to analyzing the properties of the bolus (obtained in vivo or in vitro) have been developed, measuring bolus particle size, bolus water content, and bolus rheology [12].

Studies of fragmentation and particle size distribution patterns in boluses provide information on the different comminution and agglomeration mechanisms that take place in the mouth during oral processing. A variety of methods to measure particle size distribution in the bolus have been applied, including sieving [11,12], laser diffraction [13,14], and image analysis [15]. The suitability of one method or another is determined by the specific characteristics of the food particles to be measured [13]. There is abundant information on the breakdown patterns and particle size distributions of boluses formed both in vivo and in vitro, using a wide variety of food products, based on studies considering the following groups of food: jelly, carrot, cheese, and nuts [16]; bread, cake, peanuts, and cheese [17]; peanuts, carrots, olives, mushrooms, egg, ham, chicken, cheese, and coconut [18]; nuts and vegetables [13]; bread and pasta [5]; cereal puffs and flakes [19]; and biscuits [20]. These studies have observed different fragmentation paths and a wide range of particle size distributions before swallowing, depending on the different characteristics of the food. The differences are related not only to the initial composition of the food products, but also to their initial structural and mechanical properties. In addition, in the case of in vivo studies, the results are also influenced by inter-individual differences in mastication [11,12,18,21,22].

The enzymes in human saliva include α-amylase. This enzyme hydrolyses starch in the mouth, which brings about the first step in starch degradation and in the digestion of starchy foods [23]. In many studies that have focused on the digestion of cereal-based food matrices, the sample has been ground, and the size and distribution of particles in an actual bolus during and after oral processing has normally been neglected. The study of oral starch hydrolysis is relevant as it influences the metabolic response when consuming starchy food and can also modify tastant release, altering the perception of saltiness, for instance [24]. The more disrupted the food matrix is, the higher the surface area available for enzymatic degradation by salivary and pancreatic amylase is, and the higher the blood glucose and/or postprandial insulin response is [8,25,26].

Little information is available on the relationship between the initial fragmentation and the degree of enzymatic degradation of cereal-based foods in the oral phase. One study of bread and spaghetti boluses obtained in vivo determined and compared the level of particle degradation and digestion before swallowing for each food [8]; the results highlighted the influence of oral digestion on the entire digestion process. In another recent study using white bread [27], different in vitro oral processing methods were applied to obtain boluses and their characteristics were compared with those of in vivo boluses; the kinetics of starch hydrolysis in the different boluses were also compared by performing in vitro gastrointestinal digestions.

Thus, it could be of interest to study in depth how the initial food matrix influences fragmentation and starch hydrolysis in the oral phase of digestion.

The present study aims to determine how variations in the biscuit matrix (not in composition), involving three different types of oat ingredients and the presence or absence of baking powder, affect the fragmentation pattern and the degree of oral starch hydrolysis in an in vitro approach.

## 2. Materials and Methods

### 2.1. Samples

Six oat biscuit formulations were prepared with three different oat ingredient types (flour, small flakes, and big flakes) and with or without baking powder (a mixture of diphosphate and sodium bicarbonate; Bakels aromatics, Gothenburg, Sweden). In all the formulations, the sum of the all-purpose wheat flour and the oat ingredient was kept constant, as was the proportion of the remaining ingredients except baking powder (vegetable oil, sugar, salt, and water).

The oat flour was sieved with a 2 mm mesh sieve and only the fraction that passed through the mesh was employed. The flake size was approximately 8 × 5 × 1 mm (length × width × thickness) for the small flakes and 11 × 6 × 1 mm for the big flakes.

**Figure 1:**
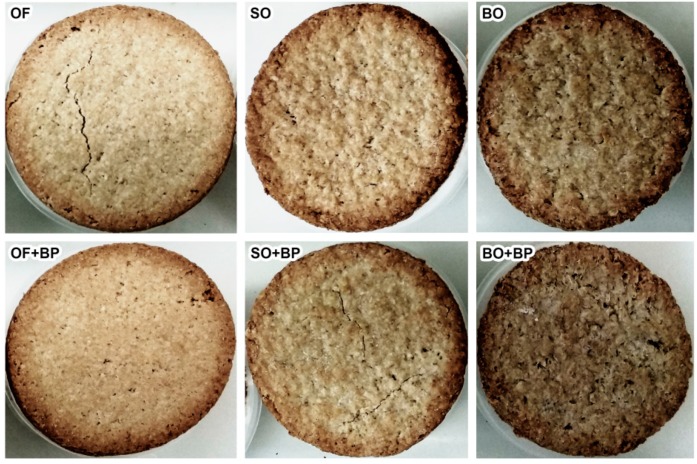
Photograph of the biscuits. OF: oat flour; SO: small oat flakes; BO: big oat flakes; +BP: with baking powder.

The ingredients (expressed in g/100 g of a 90:10 wheat flour/oat ingredient mixture) were 10 g of sugar, 20 g of vegetable oil, 5 g of salt, 66 g of water; when applicable, 0.8 g of baking powder was also added. The oat biscuits were prepared in a single stage in a Varimixer AR (Varimixer A/S, Denmark) at 120 rpm for 2 min. The dough was sheeted and molded to 5 mm thick. After molding, the dough was cut into small discs (75 mm diameter), which were placed in the oven and baked at 200 °C for 20 min. The samples were tempered to room temperature and then stored under refrigeration (4 °C) until the tests took place. Figure 1 shows the biscuits corresponding to each formulation.

### 2.2. Instrumental Texture of Samples

A three-point bending test was performed by breaking the biscuits with a TA-XT.plus Texture Analyzer equipped with Texture Exponent 32 software (version 6.0.6, Stable Microsystems, Godalming, UK). A three-point bending rig (A/3 PB) was used and the experimental conditions were as follows: supports 50 mm apart, a probe travel distance of 50 mm, a trigger force of 0.098 N, and a test speed of 0.5 mm/s [28].

Acoustic events in decibels (dB) were registered synchronically with force. A Bruel & Kjaer free-field microphone (8-mm diameter) coupled to the texture analyzer was used. The microphone was calibrated using a Type 4231 acoustic calibrator (94 and 114 dB SPL-1000 Hz) and placed near the sample (3-cm distance, 45° angle). Ambient and mechanical noise was filtered out (1 kHz high pass filter) [28].

The data acquisition rate was 500 points/s for both force and acoustic signals. All the tests were performed at room temperature in a laboratory with no special soundproofing facilities. The maximum peak force (MF) and slope to the maximum peak force (SMF) were extracted from the force–time curves. The number of sound events up to the maximum peak force (NSP) at different thresholds (>8 and >12 dB) in each curve was obtained from the sound–time curves. Five replications were conducted for each formulation.

### 2.3. In Vitro Fragmentation

The biscuits were cut into 5.0 ± 0.5 g pieces (a comfortable bite size corresponding to a quarter of biscuit) and chopped using three strokes of a hand-operated mechanical food chopper (Tescoma, Cazzago San Martino, Italy). This kitchen gadget can effect cutting events that are intended to mimic the action of teeth, so the fragmentation pattern of the samples subjected to this instrumental chopping was studied as an approach to reproducing in-mouth breakdown during the first chews. A parallel in vivo preliminary test was performed for comparison purposes: 10 subjects were asked to chew 3 times on the samples (data not shown); superficial observation of the results revealed a similar fragmentation pattern.

The particles obtained by the chopping procedure were sieved using an 841 µm mesh sieve. The fraction that passed through the sieve was weighed to calculate the percentage of particles (*w*/*w*) smaller than 0.7 mm^2^. The particle size distribution of the remaining fraction (>0.7 mm^2^) was determined by image analysis. The particles were spread out on a clean, dry, transparent glass surface (30 × 21 cm), carefully separated from each other, and their image was digitized in TIF format at 600 ppi by a scanner (Canon MP270 model K10339, Lake Success, NY, USA), using a black background. The resulting images were analyzed using Nis-Elements^®^ BR 3.2 software (Nikon, Tokyo, Japan). For this purpose, the images were binarized according to the predefined intensity threshold value, using a histogram-based segmentation process. All the objects were checked, and any unsuitable artefact (fibers and particles touching the frame) was excluded from the evaluation. The particle areas were calculated at the points where the cumulative area was 25% (a25), 50% (a50, median), and 75% (a75), as was the interquartile ratio (a75/a25). The analyses were performed in duplicate for each sample.

### 2.4. In Vitro Starch Oro-Digestion

The samples, cut into one-bite size pieces of approximately 5 g as described above, were placed in a perforated basket and submerged in 150 mL of simulated salivary fluid (SSF) [29]; the SSF (sodium bicarbonate, 5.208 g/L; potassium phosphate dibasic trihydrate, 1.369 g/L; sodium chloride, 0.877 g/L; potassium chloride, 0.477 g/L; calcium chloride dehydrate, 0.441 g/L; mucin from porcine stomach type II, Sigma, M2378, 2.16 g/L; α-amylase type VI-B from porcine pancreas, Sigma, A3176; 8.70 g/L, 200,000 units; distilled water) was adjusted to pH 6.9. All the reagents were of analytical grade.

The system was kept under constant agitation in order to achieve homogeneous sampling. An aliquot of 100 µL of the liquid was taken every 30 s up to a total time of 180 s. At each sampling time (30, 60, 90, 120, 150, and 180 s), the reaction in the aliquot was stopped quickly with 0.3 M sodium carbonate and ice, and the sample was frozen (−18 °C) for analysis on the following day.

The glucose released through starch degradation of the samples at the different points in time was quantified by the glucose oxidase-peroxidase (GOP) reaction, employing a K-GLUC enzymatic kit (Megazyme, Wicklow, Ireland). The free glucose was determined by treating the samples with the GOP reagent at 50 °C for 20 min, and the absorbance was read with a spectrophotometer at 510 nm (Biochrom Ultrospec 2100, Cambridge, UK). A glucose solution (1 mg/mL) was used as a standard. The analyses were performed in triplicate for each sample and sampling time. The degree of starch hydrolysis was calculated as the proportion of released glucose to sample weight.

### 2.5. Statistical Data Analysis

ANOVA of two factors (oat ingredient type and baking powder) and their interaction was conducted on the results of the instrumental texture analysis (maximum force, slope at maximum force, and a number of sound peaks >8 and >12 dB) and on the fragmentation parameters (percentage weight of particles with areas <0.7 mm^2^ for a25, a50, a75, and the a75/a25 interquartile ratio, and a particle size in the 0.7–5, 5–50, and >50 mm^2^ area ranges).

To study the effect of oat ingredient type and of baking powder or none on starch hydrolysis, an ANOVA of three factors (oat ingredient type, baking powder, and time) and their binary interaction was conducted.

The significance of the differences between mean values was determined by Fisher’s least significant difference (LSD) test (α = 0.05). These analyses were performed with XLSTAT statistical software (version 2019, Addinsoft, Paris, France).

## 3. Results

### 3.1. Instrumental Texture of Oat Biscuits

According to the ANOVA results, the interaction between the oat ingredient type and baking powder factors was not significant for any of texture parameters studied. The samples presented significant differences in instrumental texture parameters depending on the oat ingredient type (*p* < 0.001) (Table 1). Baking powder had a significant effect on NSP >12 dB (*p* = 0.012), but not on the other parameters (NSP > 8 dB, *p* = 0.06; MF, *p* = 0.766; SMF, *p* = 0.791).

The big oat flake biscuits required significantly less force to break them and had lower slopes at the breaking point and a higher number of acoustic events above both sound thresholds (>8 and >12 dB) than the biscuits made with oat flour. The mechanical behavior values of the small-flake biscuits were intermediate.

The presence of baking powder significantly increased the number of sound peaks in the biscuits made with oat flour. In the samples made with the other two oat ingredient types, no significant differences were found in relation to the presence of baking powder.

### 3.2. Particle Size Pattern

The weight fraction of the particles with an area of less than 0.7 mm^2^ was higher for the biscuits made with oat flour than for those made with flakes (average values 9.0%, 8.5%, and 7.2% for oat flour, small flake, and big flake biscuits, respectively), although these differences were not significant (*p* = 0.062). Baking powder had no significant effect (*p* < 0.455) on the weight fraction values.

**Figure 2:**
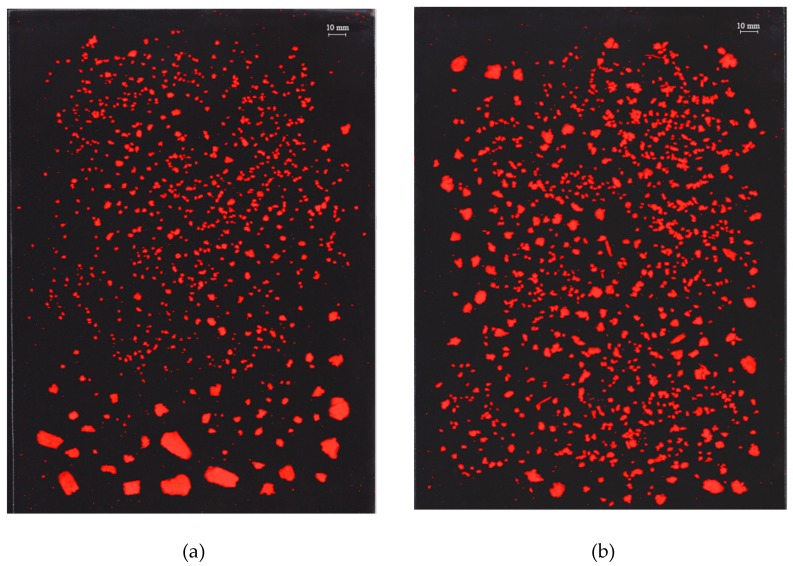
Binarized images showing the particles generated by in vitro fragmentation of samples formulated with (**a**) oat flour with baking powder and (**b**) big oat flakes with baking powder.

The number and size distribution of particles larger than 0.7 mm^2^ were analyzed through image analysis. Figure 2 shows two examples of binarized images of the fragments from different samples.

Table 2 shows particle area values at 25% (a25), 50% (a50), and 75% (a75) of the cumulative area and the interquartile ratio (a75/a25).

Parameter a25 was the only one that presented significant differences depending on oat ingredient type (*p* = 0.007). The median particle area (a50) and a75 values did not vary significantly between biscuits made with different types of oat ingredient (*p* = 0.235 and *p* = 0.071, respectively). Baking powder had no significant effect on any of the area parameter values (*p* = 0.650, *p* = 0.647 and *p* = 0.887 for a25, a50, and a75, respectively).

The interquartile ratio (a75/a25) varied between biscuits depending on the oat ingredient type (*p* = 0.001), but no differences depending on baking powder addition were found (*p* = 0.678). The a75/a25 ratio gives information on the heterogeneity of the particle size; higher values of this parameter indicate a higher degree of heterogeneity in particle size [30]. The biscuits made with oat flour and baking powder presented a significantly higher a75/a25 ratio value than the rest, which points to a more irregular fragmentation of this biscuit (Figure 2a).

**Figure 3:**
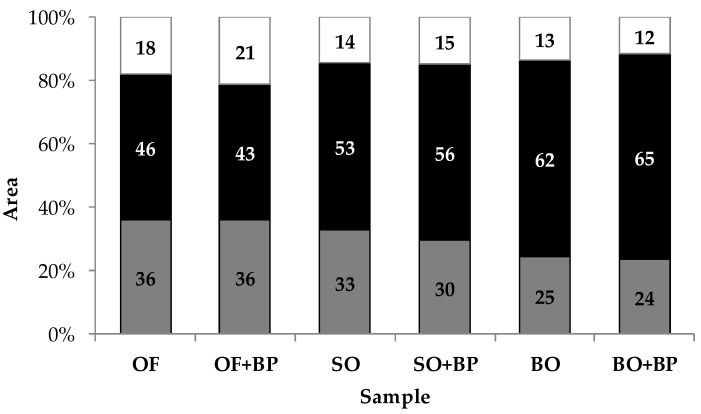
Percentage area corresponding to particles in the different size ranges. Grey: 0.7–5 mm^2^; black: 5–50 mm^2^; white: > 50mm^2^. OF: oat flour; SO: small oat flakes; BO: big oat flakes; +BP: with baking powder.

The percentage areas for each sample of particles in the different size ranges (0.7–5, 5–50, and >50 mm^2^) are shown in Figure 3.

The percentage areas of the small and middle-sized particle ranges varied significantly depending on the oat ingredient type. (*p* = 0.008 and *p* = 0.034, respectively). The biscuits made with big oat flakes presented a higher percentage of middle-sized particles (5–50 mm^2^) than the biscuits made with oat flour, which generated a higher percentage of small particles (0.7–5 mm^2^). The biscuits made with small oat flakes presented an intermediate pattern. These results indicated that the biscuits made with big flakes broke more homogeneously than those made with flour, which agrees with the a75/a25 interquartile ratio results.

### 3.3. In Vitro Starch Hydrolysis

**Figure 4:**
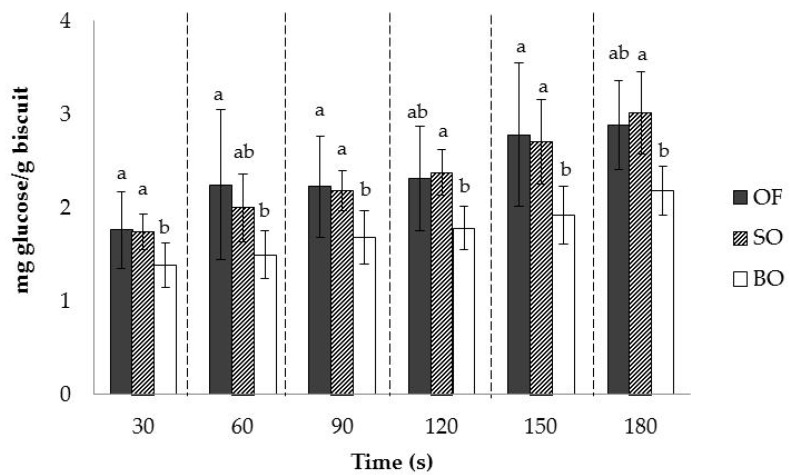
Mean values and error bars (standard deviation) of glucose released from samples over time. Mean values that do not share letters for the same period of time are significantly different (α = 0.05) according to Fisher’s least significant difference (LSD) test.

Hydrolysis of the starch in the fragmented samples was investigated (Figure 4). As expected, the amount of glucose released increased over time for all the biscuit formulations (*p* < 0.0001). The degree of starch hydrolysis over time varied significantly depending on the oat ingredient type (*p* < 0.0001) but was not affected by the addition of baking powder (*p* = 0.854). No significant interactions between oat ingredient type and time effect were detected.

Overall, the biscuits made with oat flour released more glucose than those made with big flakes. This would indicate that the oat-flour biscuit matrix was more susceptible to enzymatic hydrolysis than that of the two formulations containing big flakes.

## 4. Discussion

### 4.1. Instrumental Texture of Biscuits

The differences in instrumental texture parameter values between the samples can mainly be attributed to differences in the biscuit matrix due to the oat ingredient type used.

The only effect found for baking powder was a higher number of sound peaks for biscuits made with oat flour (OF+BP) than for the same biscuit without this addition (OF), although they were equally hard at breaking point. A biscuit has a matrix composed of a flour, sugar, and fat mass in which gas cells of different sizes and shapes are imbibed [31]. It is hypothesized that baking powder increases dough aeration by creating air cells in the matrix during baking. When a biscuit breaks, a higher number of sound emission peaks would be associated with a greater number of fragile crackling sounds, which in turn would be related to the presence of a higher number of air cells in the matrix [32]. However, this increase in sound emission due to baking powder was not detected when comparing the two pairs of biscuits made with flakes (SO vs. SO+BP and BO vs. BO+BP).

In general, the big oat flake biscuits required less force to break them and produced a higher number of acoustic events. It seems that the big oat flakes make the biscuit matrix more fragile, easier to break, and more crumbly than the oat flour biscuits. The greater effect of the big particles in the biscuit matrix probably made the effect of the baking powder more difficult to detect. It has been reported that structural irregularities and anisotropy of the structure (in the present case introduced by flakes in the biscuit dough) might have a role in fracture and crack propagation through a brittle material [33] such as a biscuit. The fractures will travel quickly resulting in sudden drops in the force curve; the fracture is then somehow inhibited and stops, only to start again as the biscuit is deformed further[34]. These drops were associated with sound events.

The mechanical behavior of the small-flake biscuits was intermediate.

Auditory cues related to sensory perceptions such as crackliness, crispness, or crunchiness are important drivers of liking in a number of foods [35]; however, in the present study the sound features were measured to relate them to the breaking characteristics of the biscuits.

### 4.2. Particle Size Pattern

The bigger oat flake biscuits fragmented into bigger pieces than the oat flour ones, which could be associated with the differences in their mechanical properties. A fragile and easily broken matrix (due to big flakes) led to a fragmentation path with bigger particles compared to the biscuits made with oat flour, while the latter had a harder matrix that yielded small particles coexisting with big ones after fragmentation. It could be hypothesized that the inhomogeneity provided by the big oat flakes created places that were preferential for initiating breaking, since cracks would start at the weakest points and structural failure could trigger cracks in nearby structural elements [28]. Along the same lines as the results of the present study, a recent study [36] using extrudates with the addition of native or fermented rye bran concluded that it is the structural attributes of the extrudates, rather than their core composition, that dictates the breakdown pattern during mastication. Another study by the same research team [37] investigated the differences in mastication (monitored by electromyography) between breads made with three different types of rye ingredients (wholemeal, endosperm, and endosperm plus gluten) and with wheat flour. The particle size distribution of the in vivo boluses indicated that wheat bread was degraded to smaller particles than rye breads during mastication, which was related to certain physical properties of the bread, such as porosity and specific volume. The authors highlighted that the initial bread matrix played a role in the degree of in-mouth breakdown. In another study that ground bread and two kinds of pasta with a modified meat grinder, the size distribution of the mechanically fragmented samples was similar to those of the corresponding in vivo boluses [5] and the differences in the degree of fragmentation of the different samples were attributed to texture differences among them. In the same way, an in vivo investigation of cereal-based (sponge cake and brioche) food boluses in elderly people [38] found that the particle size distribution in the boluses at different mastication times depended, among other factors, on the initial structure and mechanical properties of the food products.

### 4.3. In Vitro Starch Oral Hydrolysis

Some research has been conducted on in vitro methods for studying the degree of in-mouth breakdown in relation to its impact on subsequent digestion. For example, a very recent study [27] using white bread compared four different in vitro oral processing methods (cutting with a knife, cutting and grinding with a pestle, blending, and grinding) to investigate the effect of fragmentation on bolus formation and on in vitro digestion. Another study [10] subjected pastas with different compositions to four different breakdown procedures (chewing by one volunteer, grinding**/**maceration, cutting with a knife, and cutting with a knife and grinding with a pestle) followed by in vitro gastric digestion. In these two studies, the oral digestion phase was not evaluated. Other studies [39] on oral digestion of starch have indicated the potential role of salivary α-amylase activity in the perception of cooked starch.

More abundant are studies on particle sizes and distribution in in vivo boluses and their influence on digestion, although ones in which the oral phase of digestion is studied do not abound. In vivo, ready-to-swallow wheat and rye bread boluses have been compared for particle size distribution and saliva impregnation [37]; the starch hydrolysis rate in the early phase of digestion due to the salivary amylase present in the boluses was related to the differences in the structure of the breads rather than to the size of the particles in the boluses.

Particle size in the digesta after in vivo mastication and in vitro digestion [26] has been compared in breads with different compositions and textures, made with refined or wholegrain wheat and rye flour and using either a straight dough or a sourdough process. Some other factors affect the starch oral digestion. For example, the change of cooked rice grain structure in oral digestion step [40] has been showed to influence the kinetics of starch hydrolysis, which in turn is related to increases in enzyme accessibility to rice starch; such grain-scale structural changes include grain tissue damages, normally observed during the oral digestion step. It was also found that salivary amylase had an impact on the structure of hydrolysis products of cooked starches [41], and the amount and profile of lower molecular weight fractions of these hydrolysates (that are likely the substrate for subsequent hydrolysis in the gut) were different based on cooking conditions or botanical sources of starch.

In the present study, the higher susceptibility of the oat-flour biscuit matrix to the action of saliva could be explained by the presence of a higher number of small particles, which are more easily attacked by α-amylase. In addition, oat flour is obtained by milling the whole groat, so thinner oat particles are available, which can also increase its susceptibility to hydrolysis. The more broken the matrix, the higher the surface available for enzymatic degradation by salivary amylase. In turn, this effect could be related to a higher blood glucose and/or postprandial insulin response.

## 5. Conclusions

In vitro fragmentation is a practical method in oral processing studies. It avoids the concomitant variability of in vivo studies regarding individual differences and difficulty to follow up starch hydrolysis level.

In the present study, relationships between the fragmentation pattern (particle size and distribution) due to the matrix differences in the experimental oat biscuits and the incipient oral starch hydrolysis were established.

The biscuits had very similar compositions but differences were found in consideration of three different types of oat ingredient (flour, small flakes, and big flakes) and the addition or omission of baking powder. The differences in the biscuit matrix were reflected in the instrumental texture features (force and sound measurements), which in turn affected the fragmentation patterns, and could be attributed more to the effect of the oat ingredient type than to that of the baking powder. The differences in particle size distribution in the fragmented biscuits influence the degree of starch hydrolysis during the oral phase, which is more intense in samples showing a higher number of small particles.

## Figures and Tables

**Table 1:** Mean values of maximum peak force (MF), slope at maximum peak force (SMF), and the number of sound peaks (NSP) above the 8 and 12 dB thresholds, measured during fragmentation of the biscuits using a three-point bending rig.

Sample ^1^	MF (N)	SMF (N/s)	NSP (>8 dB)	NSP (>12 dB)
OF	9.6 ± 1.5 ^a,b^	7.1 ± 0.5 ^a^	12 ± 3 ^a^	1 ± 1 ^a^
OF+BP	11.3 ± 2.0 ^a^	7.6 ± 1.2 ^a^	24 ± 8 ^b^	6 ± 4 ^b^
SO	9.9 ± 0.8 ^a,b^	6.3 ± 0.9 ^a,b^	28 ± 3 ^b,c^	5 ± 3 ^b^
SO+BP	8.3 ± 0.8 ^b,c^	6.4 ± 0.9 ^a^	26 ± 6 ^b,c^	7 ± 2 ^b,c^
BO	6.8 ± 2.6 ^c^	4.9 ± 1.5 ^b,c^	34 ± 11 ^c,d^	10 ± 4 ^c,d^
BO+BP	6.3 ± 2.0 ^c^	4.1 ± 1.1 ^c^	41 ± 7 ^d^	13 ± 2 ^d^

^1^ OF: oat flour; SO: small oat flakes; BO: big oat flakes; +BP: with baking powder. Mean values in the same column that do not share letters are significantly different (α = 0.05) according to Fisher’s least significant difference (LSD) test.

**Table 2:** Particle area for 25%, 50%, and 75% of the cumulative area and the a75/a25 interquartile ratio of the biscuits after in vitro fragmentation.

Sample ^1^	a25 (mm^2^)	a50 (mm^2^)	a75 (mm^2^)a	a75/a25
OF	3.5 ± 0.7 ^a^	9.4 ± 2.8 ^a^	29.1 ± 7.5 ^a^	8.2 ± 0.6 ^a,b^
OF+BP	3.3 ± 0.1 ^a^	8.9 ± 1.5 ^a^	33.6 ± 6.3 ^a^	10.2 ± 1.8 ^a^
SO	3.8 ± 0.3 ^a,b^	9.2 ± 0.4 ^a^	23.2 ± 2.5 ^a^	6.1 ± 0.2 ^b^
SO+BP	4.1 ± 0.1 ^a,b^	10.0 ± 1.1 ^a^	22.8 ± 0.6 ^a^	5.5 ± 0.2 ^b^
BO	5.0 ± 0.6 ^b,c^	10.8 ± 1.4 ^a^	25.9 ± 2.7 ^a^	5.2 ± 0.1 ^b^
BO+BP	5.4 ± 0.8 ^c^	12.1 ± 2.5 ^a^	22.9 ± 0.9 ^a^	4.3 ± 0.5 ^b^

^1^ OF: oat flour; SO: small oat flakes; BO: big oat flakes; +BP: with baking powder. Mean values in the same column that do not share letters are significantly different (α = 0.05) according to Fisher’s least significant difference (LSD) test.
